# Perceptions and barriers to the use and training of point-of-care ultrasound among Finnish emergency physicians – a nationwide survey

**DOI:** 10.1186/s12909-024-06609-2

**Published:** 2025-01-20

**Authors:** J. Järvinen, O. Hannula, A. Meuronen, K. Mattila

**Affiliations:** 1 Faculty of Medicine, University of Turku, Turku, Finland; 2 Emergency Department, Wellbeing Services County of Central Finland, Central Finland Central Hospital, Jyväskylä, Finland; 3 Emergency Department, Wellbeing Services County of Päijät-Häme, Päijät-Häme Central Hospital, Lahti, Finland; 4 Emergency Department, Wellbeing Services County of Southwest Finland, Turku University Hospital, Turku, Finland

**Keywords:** POCUS, Emergency medicine, Ultrasound training, Training barriers, Training needs, Competency-based education, Post-graduate medical education

## Abstract

**Background:**

Point-of-Care Ultrasound (POCUS) has become integral to emergency medicine (EM) as a critical diagnostic support tool. In Finland, where EM was formally recognised as a specialty as recently as in 2013, a historical lack of systematic training for POCUS has existed. Such training has largely depended on individual initiative rather than a standardised program while many other areas of EM training have already seen the introduction of structured education. The aim of this study is to identify key factors and barriers influencing POCUS training, with the goal of improving its quality and delivery.

**Methods:**

A nationwide survey was conducted among emergency physicians, trainees, and specialists across Finnish emergency departments from late 2020 to early 2021. The survey included detailed questions on POCUS training, perceived barriers to training, experiences of the successful initiation of more structured approaches, as well as attitudes towards the integration of POCUS into clinical practice. Statistical methods for quantitative data and thematic analysis for qualitative data were used.

**Results:**

A total of 134 emergency physicians completed the survey, revealing a strong consensus among participants for several training needs. Key barriers identified include inadequate training, limited supervision, device availability, and time allocation. Notably, out of all open-ended questions, 96.5% of respondents called for the initiation of structured training programs that accommodate both foundational and advanced practitioner needs. Furthermore, hands-on training and senior support were highly valued.

**Conclusions:**

The results highlight a need for reform in POCUS training in Finland, demonstrating a need for structured, competency-based educational frameworks that align with international standards. Improvements on training infrastructure, including enhanced mentorship and increased access to ultrasound equipment, are essential enablers of such a reform.

**Clinical trial registration:**

Not applicable.

**Supplementary Information:**

The online version contains supplementary material available at 10.1186/s12909-024-06609-2.

## Introduction

Point-of-Care Ultrasound (POCUS) marks a significant advancement in emergency medicine (EM), providing a rapid, bedside evaluation tool that can improve diagnostic accuracy [1]. Physicians increasingly recognise POCUS as the “modern stethoscope” due to its numerous applications, such as the assessment of cardiac function and anatomy, FAST (Focused Assessment with Sonography in Trauma), and procedural guidance [2, 3]. Its advantages are particularly evident in the management of critically ill patients, where it improves diagnostic accuracy, shortens time to diagnosis, and accelerates therapeutic interventions — all key factors in emergency settings [3, 4].

Recognized as a distinct speciality since 2013, EM in Finland has experienced substantial growth, mirroring developments in other Nordic and European countries [5–7]. Medical physicians in Finland have traditionally received limited ultrasound (US) training at all stages of their medical education. At the undergraduate level, programs integrating POCUS training are still globally uncommon, although there is a noticeable trend toward incorporating POCUS into medical curricula [3, 8, 9]. As individuals transition into post-graduate speciality training, US training becomes more tailored, focusing primarily on field-specific needs. However, the advantages of POCUS largely depend on the operator’s competence, underscoring the necessity for comprehensive training programs [3]. In 2020, Finland introduced a competency-based specialist training model, transforming training by focusing on systematic and structured teaching through Entrustable Professional Activities (EPA) [7, 10, 11]. In its current, early form, the EM physician model does not incorporate a POCUS-based EPA. The program dedicates only one out of 26 professional qualifications to POCUS [12].

Prior research has shown that attitudes toward POCUS adoption, perceived barriers to its effective use, and variations in training quality have an impact on the use of POCUS in clinical practice [13]. Research has also shown that fear of misinterpreting findings and apprehension concerning the medical significance of obtained results characterise attitudes towards POCUS use [14, 15].

Negative attitudes, insufficient faculty training, and inadequate senior supervision and feedback can limit POCUS adoption and usage [16]. In addition, insufficient practice time, administrative support, US machine access, and exam quality assessment are other barriers [17–19]. Depending on the country, speciality, supervisor attitudes, and in-house or third-party training, training programs vary greatly in content and quality. Short, intensive courses and more comprehensive programs that focus on practical skills or theoretical knowledge are available [20–22]. Addressing POCUS barriers is essential for identifying and closing training and practice gaps and ensuring emergency physicians are proficient, confident, and accurate in varied clinical circumstances. Notably, no prior research has explored POCUS barriers and attitudes in Finland.

The aim of this study is to identify key factors influencing POCUS training, with the goal of improving its quality and delivery. To accomplish this we carried out a nationwide survey among Finnish EM trainees (also referred to as residents) and consultant-grade specialists (also referred to as specialist) to explore the attitudes and perceived barriers toward POCUS.

## Materials and methods

### Study design, participants, and execution

A cross-sectional survey study was done among EM physicians across Finland. The survey was first conducted as a paper-based pilot in fall 2020 with EM trainees and specialists from four hospitals, followed by an expanded online survey targeting all EM professionals nationwide in early 2021.

The survey was designed by a group of EM specialists with expertise in POCUS, with assistance from a statistician experienced in survey development. The total study population included 253 physicians, of whom 197 were trainees and 56 were consultant-grade specialists. Several platforms were utilized to disseminate the survey, including the Finnish Society of Emergency Medicine’s membership roster. Participation was voluntary, and survey completion constituted informed consent. No identifiable respondent data was collected. The Pirkanmaa Hospital District Ethics Committee approved the study.

A more detailed description of the survey design, participants, and execution is described in the previous article [23].

### Methods

Respondents reviewed various training methods, including bedside instruction, individual practice, structured sessions, and external courses, reporting time spent and perceived benefits using Likert scales. Participants also rated factors influencing POCUS integration into their work, such as equipment availability, confidence in findings, and feedback from seniors. Mean numerical rating scales (NRS) were analyzed to identify key trends. The complete survey form is included in the appendix.

Thematic analysis was conducted to systematically review, code, and categorize responses to open-ended questions into themes and sub-themes [24]. Thematic analysis, done by the first author (JJ), identified the main themes of training needs, mentorship and guidance, equipment access, and barriers. The category of training needs was split into five sub-themes: structured training, hands-on training, routine practise, specialised sessions and time allocation. Themes were accompanied with the most frequently cited representative quotes. The analysis of theming was performed on the entire group of respondents as well as separately on the consultant-grade respondents and trainees.

The quantitative data obtained was also categorised into trainees and specialists to emphasise the similarities and contrasts in their demands and experiences.

The statistical analyses were performed using SPSS Statistics 29.0 software (IBM Corp., Armonk, NY). The study examined the statistical significance between trainees and specialists in non-parametric samples using the Mann-Whitney test. The Chi-Square test was used to analyze binomial variables. A p-value less than 0.05 was deemed statistically significant for all analysis.

## Results

A total of 96 trainees and 38 consultant-grade emergency medicine specialists throughout Finland answered the survey, providing a response rate of 53% (Table 1). Out of 134 respondents, 57 provided an open-ended answer (43%).

**Table 1:** Respondent demographics

Characteristic	Total	Junior trainees	Senior trainees	Specialists
Number of participants	134	56 (42%)	35 (26%)	38 (28%)
Age (Years, SD)	36 (6.0)	32 (4.5)	37 (5.5)	39 (4.3)
Sex				
- Male	73 (55%)	30 (54%)	17 (49%)	21 (58%)
- Female	59 (44%)	26 (46%)	18 (51%)	15 (42%)
Work occupation				
- University hospital	49 (37%)	18 (33%)	15 (44%)	14 (37%)
- Secondary hospital	69 (53%)	32 (58%)	15 (44%)	20 (53%)
- Other	13 (10%)	5 (9%)	4 (12%)	4 (11%)
Uses ultrasound in their clinical work	126/132 (94%)	51/55 (93%)	34/35 (97%)	37/37 (100%)

Due to the varying numbers of partial non-responders and rounding up, all of the percentages might not equal 100%

### Training needs

A substantial consensus among participants (96.5% of open-ended answers) underscores the necessity for multiple aspects of training, with 31.6% emphasizing the importance of structured, goal-oriented, and systematic approaches (Table 2). Trainees wanted to see systematic and scheduled training, while specialists proposed more speciality-specific training and national-level training for instructors (Table 3). The impact of structured training on the use of POCUS was considered high, as shown in Table 4, as both groups rated it at a mean NRS of 4 or higher. Specialists engage in structured workplace training more frequently each year (mean 2.60 days for specialists vs. 1.89 days for trainees). Both groups (mean NRS trainees 4.08, specialists 3.97) rate courses outside the workplace highly.

**Table 2:** Thematic analysis of training needs and challenges

Theme	Sub-theme	*n* (%) out of 57	Representative quotes
Training Needs (*n* = 55, 96.5%)	Structured Training	18 (31.6%)	“POCUS training could be more structured and goal-oriented.” “Systematic schooling from the beginning ensures comprehensive skill development.”
	Hands-on Training	12 (21.1%)	“More practical work and training with US.” “Hands-on stuff, learning by doing when there is sufficient background knowledge.”
	Routine Practice	11 (19.3%)	“POCUS should be used routinely in patient assessments to improve clinical outcomes and practitioner skills.” “Integrating POCUS assessment into standard patient evaluations ensures its regular application and skill reinforcement.”
	Specialised Sessions	8 (14.0%)	“A radiology rotation should be mandatory.” “More targeted training sessions, such as courses or rotations specifically in POCUS.”
	Time Allocation	6 (10.5%)	“There is no time to practice in practical work.” “Allocate time for teaching to ensure consistent skill application.”
Mentorship and Guidance		15 (26.3%)	“There is currently no opportunity to practice under more skilled supervision.” “More senior supervision, feedback, and direct mentoring in using POCUS are crucial for effective learning.”
Equipment Access		9 (15.8%)	“More portable devices to lower the threshold for use.” “Increasing the number of portable ultrasound devices in the emergency department to facilitate practice.”

The table presents a breakdown of the common themes identified in the training and education of POCUS through open-ended answers. The representative quotes reflect the overall feedback from both trainees and specialists, emphasising the key areas for improvement and the number of responses analysed (*n* = 57). Proportions exceed 100% due to overlapping themes where an individual respondent mentioned several needs and challenges

**Table 3:** Comparative perspectives on POCUS Training challenges and needs

Theme	Sub-theme	Trainees’ perspectives	Specialists’ perspectives
Barriers	Knowledge	- Frequent mentions of a lack of foundational knowledge.	- Less emphasis, possibly due to higher experience levels.
	Time Constraints	- Expressed frustration over insufficient time for practice due to workload.	- Concerns about allocating time for mentoring amidst other responsibilities.
Training Needs	Structured Training	- Desires more organised, systematic schedules.	- Focus on integrating advanced, specialty-specific training. - National-level training for instructors
	Hands-on Training	- Hoping for more interactive, simulation-based learning.	- Use of simulation and other modern educational tools to enhance learning
	Routine Practice	- Need for regular, embedded training in daily routines.	- Emphasis on ensuring routine training is practical and applicable.
Mentorship and Guidance		- A need for more accessible, consistent supervision and feedback.	- Respondents proposed more structured mentorship, especially in resource-limited settings.
Equipment Access		- Calls for better US device availability to enhance hands-on practice.	- Answers emphasised ensuring every trainee has personal access to US devices for skill retention and practice.

The table presents the summarised key perspectives of trainees and specialists on the themes which arose from the survey’s open-ended answers. Each theme is explored to highlight the key needs and suggestions from both groups

**Table 4:** Formal training and impact on practical work

Form of ultrasound training	Days/Year (trainees)	Days/Year (specialists)	Impact on use of POCUS (mean NRS) (trainees)	Impact on use of POCUS (mean NRS) (specialists)
Lecture teaching	1.67	1.80	3.94	3.50
Structured workplace training	1.89	2.60	4.25	4.03
Phantom/tissue model training	0.67	0.62	3.51	3.21
Ultrasound course(s) outside workplace	1.26	1.00	4.08	3.97

The table presents formal US training methods and educational resources on the application of POCUS in practical work

### Hands-on and routine practice

**Fig. 1:**
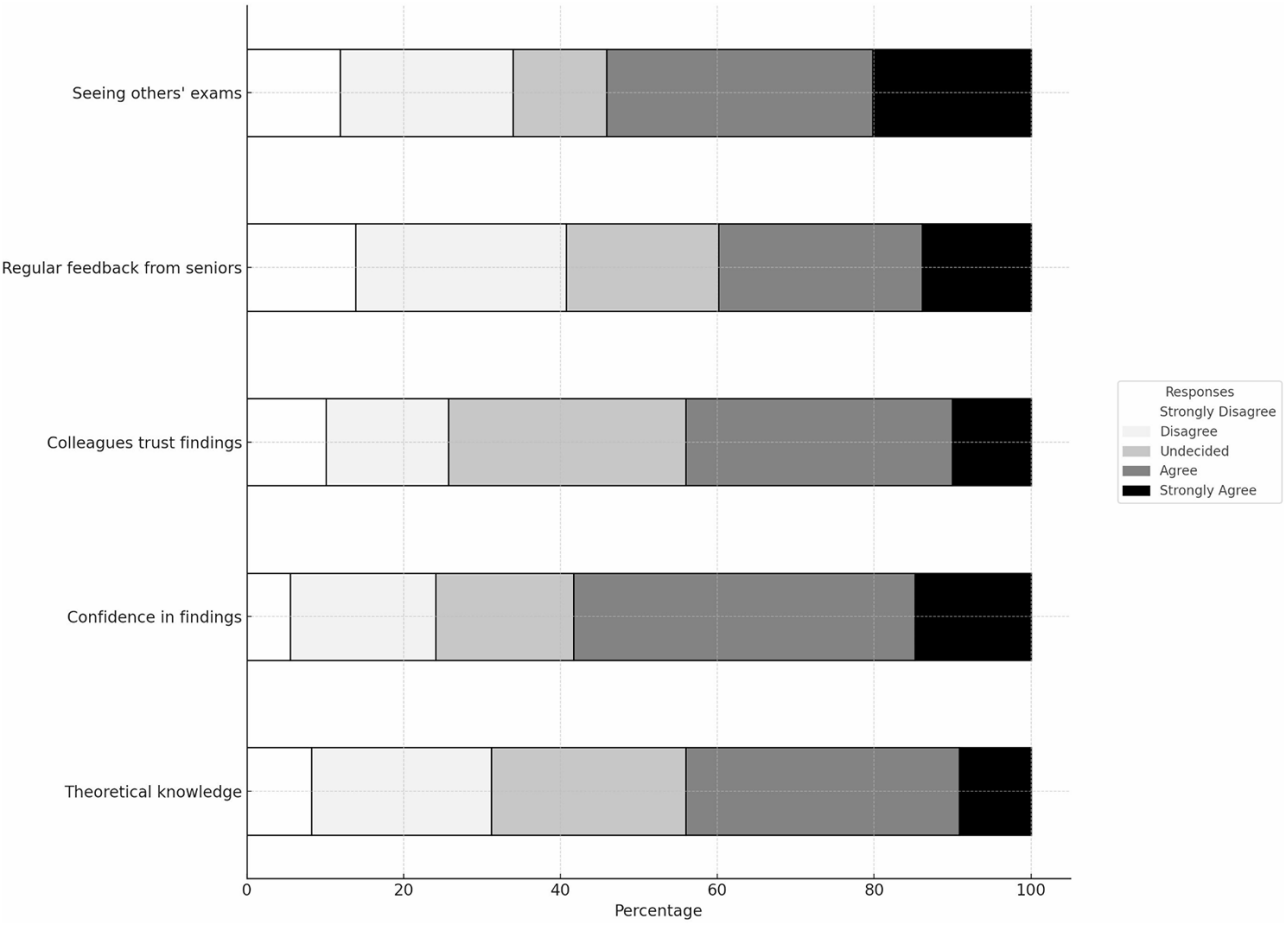
Illustrates the promoting factors towards the use of US in their current work roles, based on responses to a 5-point Likert scale. No significant differences were found between trainee and specialist groups

As detailed in Table 2, many respondents highlighted the importance of hands-on training and routine practice with these subthemes accounting for 40.4% (hands-on training 21.1%, routine practice 19.3%) of all responses regarding training needs. Hands-on training was proposed with modern educational tools, with possibilities for more interactive, simulation-based learning opportunities, while ‘routine practice’ refers to the regular integration of training and use of US into daily clinical tasks (Table 3). Over 50% agreed (NRS 4 or better) that seeing other colleagues do US exams promotes the use of POCUS (Fig. 1). Overall, trainees and specialists spent an average of 4.68 and 5.23 respective hours per month on independent US training, and both rated the impact of this practice to be high (mean NRS of 4.33 for both groups) (Table 4).

### Mentorship and guidance

Recognition of the need for improved mentorship was evident in 26.3% of open-ended answers (Table 2). Trainees reported a frequent need for more accessible and consistent supervision, reflecting their reliance on guidance for skill acquisition (Table 3). Table 5 reflects this, with trainees rating a senior bedside exam and training under supervision as having a high impact (mean NRS 4 or better) on their US use. Figure 1 reflects the appreciation of senior feedback. Specialists advocated for structured and adaptable mentorship programs, especially in settings limited by resources (Table 3).

**Table 5:** Informal training and impact on practical work

Form of ultrasound training	Hours/Month (trainees)	Hours/Month (specialists)	Impact on use of POCUS (mean NRS) (trainees)	Impact on use of POCUS (mean NRS) (specialists)
Reading textbooks and scientific articles	0.95	1.16	3.36	3.52
Studying the contents of websites such as blogs, as well as listening to podcasts	1.37	1.50	3.69	3.71
Bedside teaching by a senior	1.34	1.10	4.30	3.84
Ultrasound training under supervision	0.95	0.93	4.33	3.86
Ultrasound training independently	4.68	5.23	4.33	4.33

This table presents the effects of informal US training methods and educational resources on the application of POCUS in practical work comparing trainees and specialists

### Barriers

**Fig. 2:**
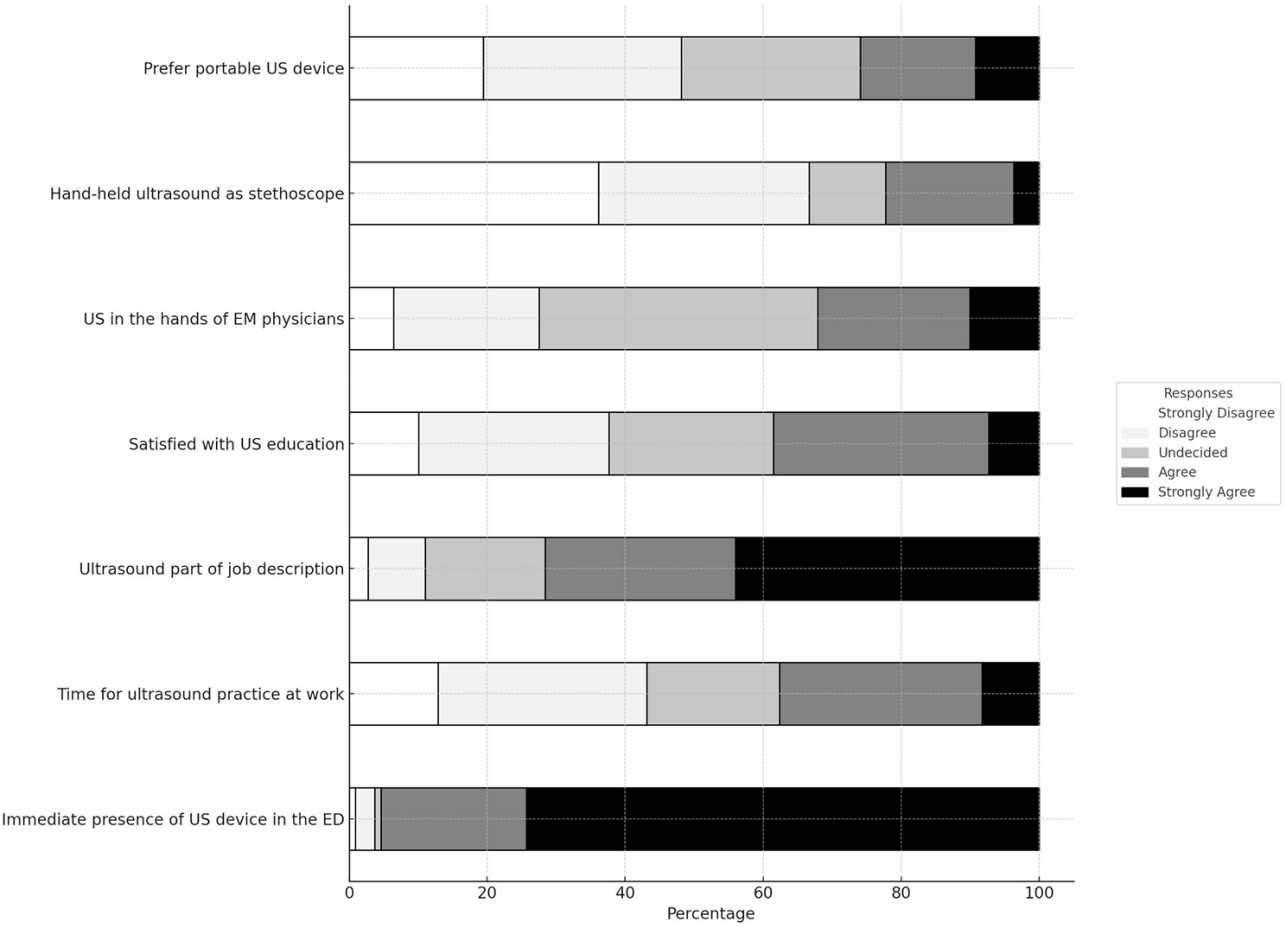
Illustrates the attitudes and perceptions of respondents towards the use of US in their current work roles, based on responses to a 5-point Likert scale. No significant differences were found between trainee and specialist groups

Both groups identified knowledge gaps and time constraints as barriers, with trainees in particular struggling with a lack of foundational knowledge and the challenge of balancing heavy workloads with training opportunities (Table 3). The majority of the participants agree that adequate knowledge promotes the use of POCUS (Fig. 1). Specialists focus more on the logistical challenges of integrating mentoring into their schedules, as their experience alleviates the burden of basic knowledge gaps (Table 3). We noted concerns about time allocation for practical training, which impacted 10.5% of open-ended answers (Tables 2 and 3) and reflected attitudes toward US use (Fig. 2).

### Equipment access

Many respondents felt that the good availability of devices increased the use of POCUS in Fig. 2; the open-ended answers emphasised the challenging accessibility of these devices in Table 2. Both groups expressed the belief that the portability of US devices would enhance accessibility within EM departments. Trainees expressed a need for the greater availability of US devices during shifts to facilitate hands-on practice, while specialists suggested personal device access to ensure skill retention and proficiency (Table 3).

## Discussion

This study investigates the training needs, barriers, and current practices of POCUS used by Finnish emergency physicians. In this study, we identify several key themes regarding training needs, barriers, and attitudes toward POCUS within the field of EM in Finland.

According to our data, the most popular themes concerning training needs among all EM physicians are the vast need for structured training and more hands-on training, as well as the need to implement POCUS in everyday practice. Other common barriers include a lack of mentorship and guidance, as well as inadequate access to US devices. Specialists and residents share common themes with variation in details. Perceptions, attitudes, and factors promoting US use are identical between trainees and specialists here.

A combined total of over 40% of all training needs are attributed to hands-on training and routine practice, and over 30% emphasise the need for structural training in this study. Previous studies have identified the lack of training and the absence of a formal curriculum as major training needs [14, 25]. In previous studies, simulation-based learning has been found to be a highly effective method in medical education, providing opportunities for hands-on practice in a controlled environment [26, 27]. As suggested by trainees in this study, incorporating simulation-based learning in US training curriculum could be an example of relatively straightforward modality to enhance hands-on training.

A significant portion of both trainees and specialists report a clear preference for US technology as part of their regular clinical practice. Such independent training is critical to the learning process, allowing learners to apply theoretical knowledge in practical settings, which enhances retention and skill proficiency [28, 29]. However, trainees report that heavy workloads often prevent routine independent practice, despite their preference for independent POCUS training; other settings have documented similar reports [30, 31]. It is not surprising that this theme emerges as a challenge in busy EM departments. To counter this barrier, well-structured and adequately resourced training programs that can allocate dedicated time for practicing and training POCUS are needed.

Our data supports the need for improved mentorship in POCUS training, with over a quarter of the open-ended responses underscoring it. Several trainees expressed a need for more accessible and consistent supervision, as evidenced by their high appreciation for following a senior’s bedside exam and receiving training under supervision. This is in line with previous studies [14, 25]. This indicates a significant appreciation for direct mentorship, which enhances their POCUS usage and overall competence [32, 33].

These findings, and correcting them, support the pedagogical shift toward competency-based medical education, which promotes experiential learning where learners actively integrate knowledge and practice new skills. Previous studies have demonstrated the benefits of experimental learning, beginning at the undergraduate level of training [34]. In Shokoohi’s study, preclinical medical students improved significantly in FAST image interpretation during a one-year period with an experimental learning model [35].

Lastly, the respondents highly value portability and immediate access to US devices, yet find them lacking. This highlights the ongoing challenge of ensuring that all training facilities are well-equipped. Although the overall availability and accessibility of US equipment in EM departments has improved over the last decade [36–38], this study reveals that there is still room for improvement.

### Strengths and limitations of this study

The mixed methods design of this study was chosen to capture a comprehensive understanding of EM physicians’ attitudes toward POCUS and the barriers they encountered. This approach allowed for the collection of a wide range of insights, enabling the study to capture versatile feedback and address potential gaps that the survey designers might not have anticipated. The inclusion of predefined options provided a structured framework that helped mitigate the subjective interpretations often associated with open-ended questions, thereby enhancing the overall objectivity and quality of the data.

This study has several limitations. The response rate for our survey was 53%. While it is considered favourable compared to similar studies [13, 18, 39, 40], nearly half of the targeted population — individuals engaged in clinical practice — failed to respond. This could potentially introduce generalisation and non-response bias. Despite studies suggesting that non-response bias does not significantly impact survey research outcomes, this factor could still influence the validity of our findings [41]. The retrospective nature of the survey may have introduced some inaccuracy in recalling details, such as the amount of US training received.

An additional limitation relates to the methodological approach of this study. The data interpretation during the coding and theming process may introduce inconsistencies, potentially biasing the conducted thematic analysis [42]. We employed peer debriefing and review processes throughout the research to counter these effects and enhance the reliability and validity of the thematic outcomes [43].

## Conclusions

This study elucidates evolving trends and imperatives in POCUS training within EM. It reveals a need for structured, flexible training programs tailored to both novice and advanced practitioners. We identified key barriers such as inadequate training, limited supervision, device availability, and time, which clearly indicate areas where we could enhance the current training curriculum. The introduction of structured training models is a transformative step in Finnish POCUS educational practices, highlighting the necessity for well-organized training programs complemented by mentorship and access to US devices. The development of a European curriculum for POCUS training by European Society For Emergency Medicine (EUSEM) and the potential creation of an EPA for POCUS training would be useful steps towards standardising training and making it a mandatory part of residency programs, which would ensure consistent training quality across Europe. We need to conduct a follow-up study in the coming years to evaluate the implementation of necessary changes in POCUS training and their impact on the desired improvements in training quality.

## Electronic supplementary material

Below is the link to the electronic supplementary material.

Supplementary Material 1

## Data Availability

The data that support the findings of this study are available from the corresponding author upon reasonable request.
